# Dissecting the contribution of human chromosome 21 syntenic regions to recognition memory processes in adult and aged mouse models of Down syndrome

**DOI:** 10.3389/fnbeh.2024.1428146

**Published:** 2024-07-10

**Authors:** Tara Canonica, Emma J. Kidd, Dorota Gibbins, Eva Lana-Elola, Elizabeth M. C. Fisher, Victor L. J. Tybulewicz, Mark Good

**Affiliations:** ^1^School of Psychology, Cardiff University, Cardiff, United Kingdom; ^2^School of Pharmacy and Pharmaceutical Sciences, Cardiff University, Cardiff, United Kingdom; ^3^Francis Crick Institute, London, United Kingdom; ^4^Department of Neuromuscular Diseases, UCL Queen Square Institute of Neurology, London, United Kingdom

**Keywords:** down syndrome, mouse models, recognition memory, object tasks, aging, hippocampus, glutamate receptors

## Abstract

**Background:**

Trisomy of human chromosome 21 (Hsa21) results in a constellation of features known as Down syndrome (DS), the most common genetic form of intellectual disability. Hsa21 is orthologous to three regions in the mouse genome on mouse chromosome 16 (Mmu16), Mmu17 and Mmu10. We investigated genotype-phenotype relationships by assessing the contribution of these three regions to memory function and age-dependent cognitive decline, using three mouse models of DS, Dp1Tyb, Dp(17)3Yey, Dp(10)2Yey, that carry an extra copy of the Hsa21-orthologues on Mmu16, Mmu17 and Mmu10, respectively.

**Hypothesis:**

Prior research on cognitive function in DS mouse models has largely focused on models with an extra copy of the Mmu16 region and relatively little is known about the effects of increased copy number on Mmu17 and Mmu10 on cognition and how this interacts with the effects of aging. As aging is is a critical contributor to cognitive and psychiatric changes in DS, we hypothesised that ageing would differentially impact memory function in Dp1Tyb, Dp(17)3Yey, and Dp(10)2Yey, models of DS.

**Methods:**

Young (12-13 months and old (18-20 months mice Dp1Tyb, Dp(17)3Yey and Dp(10)2Yey mice were tested on a battery of object recognition memory test that assessed object novelty detection, novel location detection and associative object-in place memory. Following behavioral testing, hippocampal and frontal cortical tissue was analysed for expression of glutamatergic receptor proteins using standard immunoblot techniques.

**Results:**

Young (12-13 months and old (18-20 months mice Dp1Tyb, Dp(17)3Yey and Dp(10)2Yey mice were tested on a battery of object recognition memory test that assessed object novelty detection, novel location detection and associative object-in place memory. Following behavioral testing, hippocampal and frontal cortical tissue was analysed for expression of glutamatergic receptor proteins using standard immunoblot techniques.

**Conclusion:**

Our results show that distinct Hsa21-orthologous regions contribute differentially to cognitive dysfunction in DS mouse models and that aging interacts with triplication of Hsa21-orthologous genes on Mmu10.

## 1 Introduction

Down syndrome (DS) is a neurodevelopmental disorder caused by trisomy of chromosome 21 (Hsa21) and estimated to affect 12.8 of every 10,000 live births (Antonarakis et al., 2020). In addition to physical characteristics, such as a distinctive skull shape, individuals with DS have intellectual disability and present with learning and memory impairments. For example, people with DS are less able than neurotypical individuals to discriminate between novel and previously studied images, three-dimensional objects, landmarks within virtual navigation environments, and in associative visuospatial memory (Vicari et al., 2005; Lavenex et al., 2015; Purser et al., 2015; Clark et al., 2017). In DS, temporal lobe volume is greatly reduced compared to neurotypical individuals (Takashima et al., 1994; Pinter et al., 2001; Guidi et al., 2008), with MRI studies reporting a ~30% volume reduction in the hippocampus (HPC), the primary structure supporting declarative recognition and spatial memory functions (Barker and Warburton, 2011). Morphological alterations of the HPC are already present in DS fetuses, persist through adulthood and are exacerbated with age (Takashima et al., 1994; Guidi et al., 2008; Fukami-Gartner et al., 2023).

**Figure 1:**
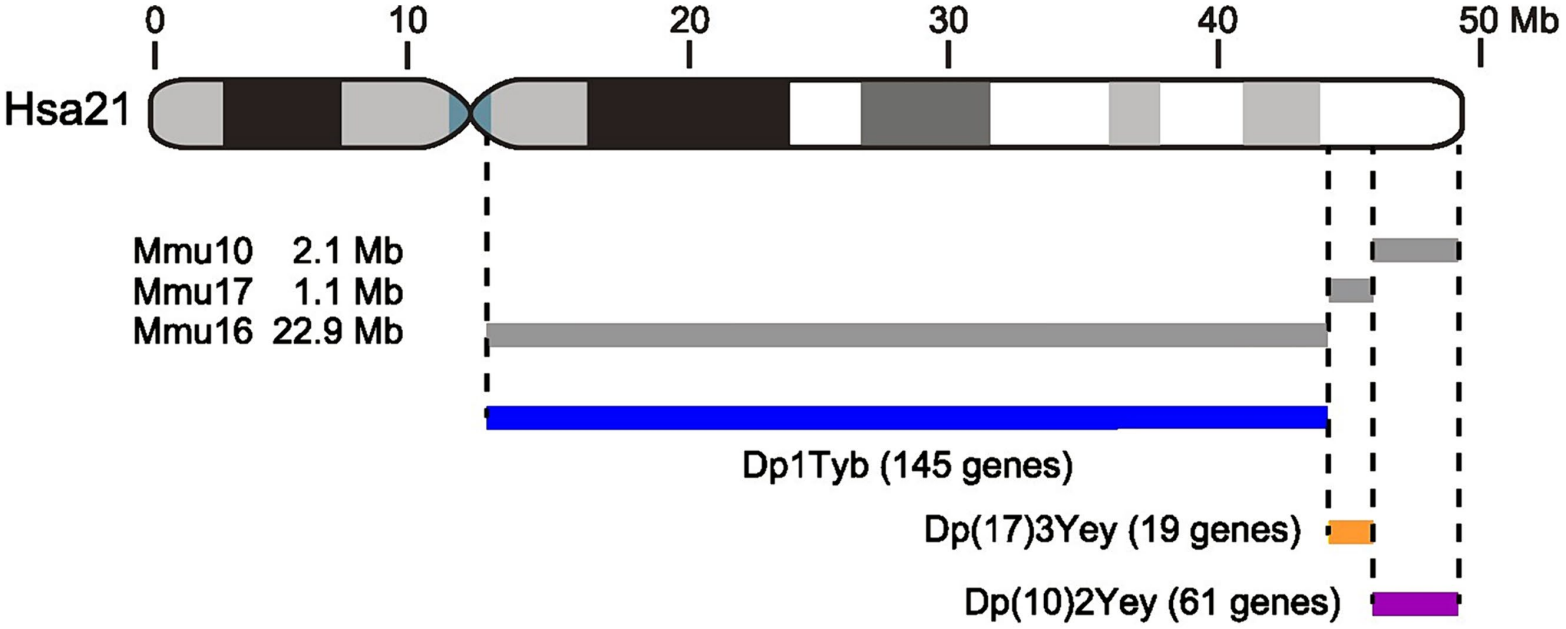
Schematic of Hsa21 and the three mouse models used in this study. Diagram of Hsa21 at the top showing short and long arms and the centromere (blue). Gray and black boxes indicate cytogenetic bands. Length of the chromosome is indicated in Mb. Gray lines below Hsa21 indicate regions of orthology on Mmu16, Mmu17, and Mmu10 and their DNA lengths. Note that the short arm of Hsa21 contains largely repetitive DNA and does not have known orthology to the mouse genome. The three mouse models, Dp1Tyb, Dp(17)3Yey, and Dp(10)2Yey have an extra copy of each of these three Hsa21-orthologous regions of the mouse genome. Numbers of protein coding genes are indicated in parentheses, and were calculated as described in the Methods.

Although several Hsa21 genes have been implicated in cognition (e.g., *DYRK1A*, *APP*; Dowjat et al., 2007; Wiseman et al., 2018; Tosh et al., 2021), the link between aberrant dosage of Hsa21 genes, memory dysfunction and cognitive decline remains to be fully understood. Hsa21 is ~1.5% of the human genome (Hattori et al., 2000), with 235 protein-coding genes, 441 non-coding RNA genes and 188 pseudogenes currently identified (Ensembl release 105, Howe et al., 2021). The long arm of Hsa21 is evolutionarily conserved in the mouse genome over syntenic regions on three mouse chromosomes: Mmu16 (*Lipi*-*Zbtb21*, ~28 Mb), Mmu17 (*Abcg1*-*Rpr1b*, ~1.5 Mb) and Mmu10 (*Prmt2-Pdxk*, ~3 Mb) (Figure 1; Gupta et al., 2016). Mouse strains with an extra copy of these Hsa21-orthologous regions of the mouse genome have been generated to assess their contribution to DS phenotypes. These mouse models of DS recreate physical and cognitive DS-like features and have been used to identify regions containing genes responsible for specific DS phenotypes (Herault et al., 2017). Most research has focused on models with an extra copy of the Mmu16 region, since this region contains the largest number of Hsa21-orthologous genes. In contrast, relatively little is known about the effects of increased copy number of the Hsa21-orthologous regions on Mmu17 and Mmu10. For example, memory function has been primarily investigated in the Dp1Tyb and the related Dp(16)1Yey strains that have an extra copy of the same Hsa21-orthologous Mmu16 region; trisomy of these regions results in recognition memory deficits, slower decision making but accurate spontaneous alternation, and deficits in hippocampal long-term potentiation (LTP) and hippocampal theta high gamma phase-amplitude coupling (Yu et al., 2010a,b; Souchet et al., 2014, 2019; Aziz et al., 2018; Chang et al., 2020; Lana-Elola et al., 2021). In contrast, Dp(10)2Yey mice (which are duplicated for the Hsa21-syntenic region of Mmu10) show reduced spontaneous alternation at 3, 6 and 9 months of age and reduced hippocampal theta (low gamma) phase coupling. Dp(17)3Yey mice (which are duplicated for the Hsa21-syntenic region of Mmu17) showed no behavioral or abnormal hippocampal theta-phase dynamics relative to wildtype (WT) mice (Chang et al., 2020). Aging is a critical contributor to cognitive and psychiatric changes in DS. DS patients show the key neuropathological features of Alzheimer disease (AD) by the age of 40 and have a high risk of dementia by the age of 60 (Lott and Dierssen, 2010; Cannavo et al., 2020). To date, few studies have examined behavioral changes in mouse models of DS as a function of age.

In the present study cognitive function was examined in Dp1Tyb, Dp(17)3Yey, and Dp(10)2Yey mouse models of DS, at 12–13 and 18–20 months of age. We used a battery of object-based recognition memory tasks to assess brain networks sensitive to aging (Canatelli-Mallat et al., 2022) and critical for novelty detection and spatial organization memory. The battery of recognition tests was selected to minimize perceptual and motoric differences between tasks that discriminate between different attributes of object, location and associative memory. The results reveal that Dp1Tyb and Dp(10)2Yey mice displayed, respectively, an age-independent and an age-dependent change in recognition memory. In contrast, object memory function was unaffected at both ages in Dp(17)3Yey mice. Activity of glutamate receptors is a requirement for normal synaptic potentiation and for intact performance in object-based recognition memory tasks (Barker et al., 2006; Barker and Warburton, 2015; Benn et al., 2016). In line with the observed memory performance, hippocampal glutamate receptor expression was altered in Dp1Tyb and aged Dp(10)2Yey mice, but unaltered in Dp(17)3Yey mice. Our findings show that specific Hsa21 syntenic regions in the mouse contribute to distinct DS-like behavioral phenotypes and may interact with brain aging.

## 2 Methods

### 2.1 Animals

C57BL/6 J.129P2-Dp(16Lipi-Zbtb21)1TybEmcf (Dp1Tyb); C57BL/6 J.129S7-Dp(10Prmt2-Pdxk)2Yey/J [Dp(10)2Yey] and C57BL/6 J.129S7-Dp(17Abcg1-Rrp1b)1Yey [Dp(17)3Yey] male mice were used in these studies, all of which have been previously described (Yu et al., 2010a,b; Lana-Elola et al., 2016). All strains were bred at the Francis Crick Institute, backcrossed for at least 10 generations to C57BL/6 J and maintained in separate colonies as hemizygous mutants. Age-matched wild-type littermates were used as controls.

All experiments were performed in accordance with the United Kingdom Animal (Scientific Procedures) Act 1986 and under License from the UK Home Office. Dp1Tyb, Dp(17)3Yey, and Dp(10)2Yey cohorts of male mice were transferred to Cardiff University at 3 months of age for behavioral analysis. Mice were housed in groups of 2–5 age-matched animals in individually ventilated cages (IVC) under controlled environmental conditions (24–25°C, 50–60% humidity, 12 h light/dark cycle) with *ad libitum* access to food and water. All behavioral experiments were undertaken blind to genotype and decoded after experimental analysis.

Numbers of protein-coding genes in different mouse strains were determined using the Biomart function in Ensembl on mouse genome assembly GRCm39, filtering for protein-coding genes, excluding three genes on Mmu16: ENSMUSG00000116933 which is a partial transcript for *Atp5o* (ENSMUSG00000022956), *Gm49711*, which is an alternatively spliced form of *Mrps6*, and *Gm49948* which is a fusion transcript of some exons from *Igsf5* and *Pcp4*. Note that the numbers of duplicated coding genes in the Dp1Tyb strain have changed since our original publication (Lana-Elola et al., 2016), due to changes in gene annotation.

### 2.2 Elevated plus maze test

The test was run in an 86 cm (L) × 86 cm (W) × 74 cm (H) × 6 cm (internal W) plus-shaped maze consisting of two closed arms and two open arms, with a small, neutral center area (6 × 6 cm^2^). The animal was placed in the center of the maze and allowed to explore the environment for 5 min while its locomotor behavior was tracked with Ethovision XT 13 (Noldus Information Technology) and used to determine anxiety and activity levels. The percentage of time spent in open arms during the entire trial duration was calculated as follows*: (seconds in open arms/(seconds in open arms + seconds in closed arms) × 100%).*

### 2.3 Recognition memory tasks

**Figure 2:**
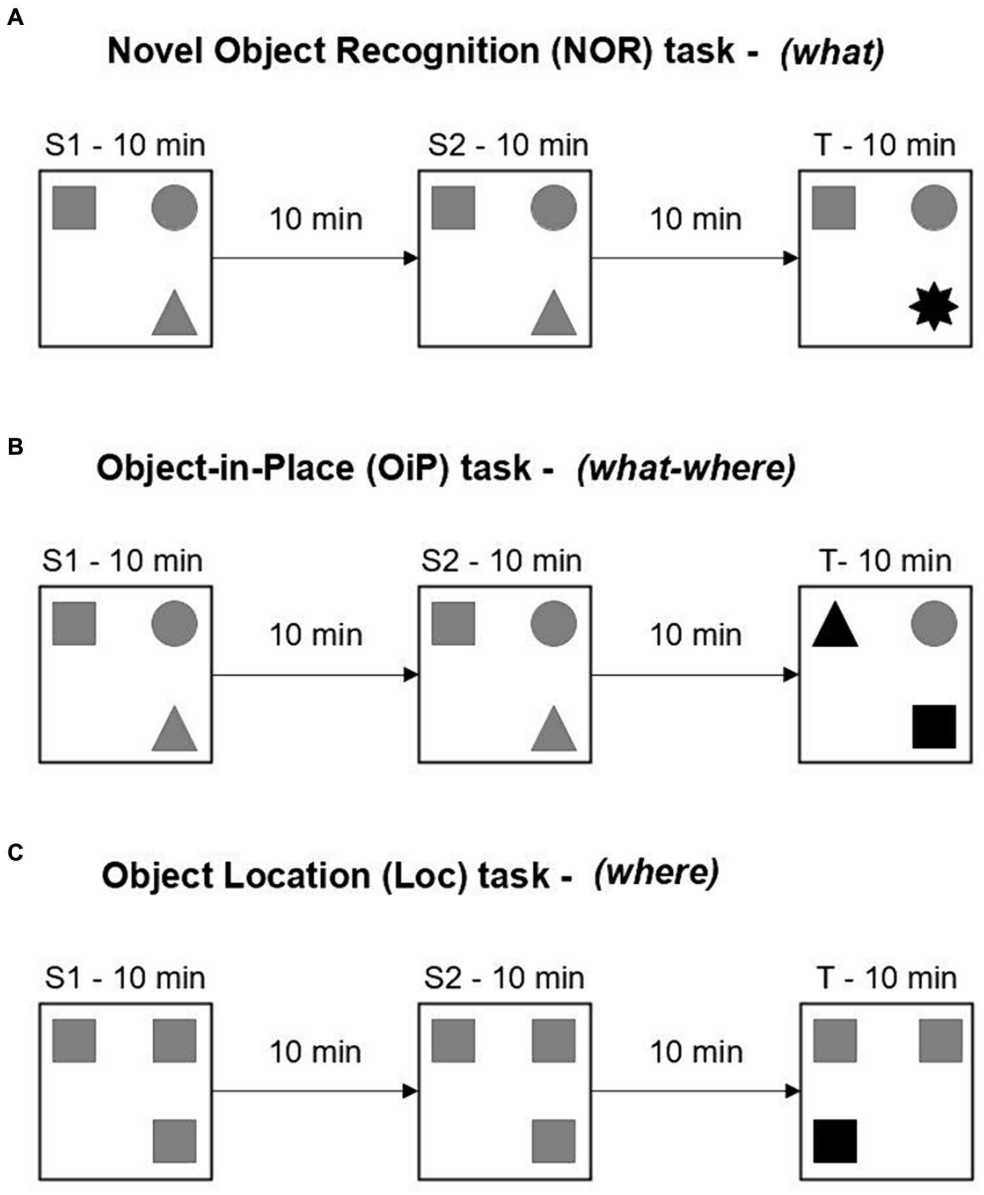
Design of recognition memory tasks. **(A–C)** Three different recognition memory tasks used in this study. Different symbols represent different objects. Target objects are in black. Each phase lasted 10 min and was separated by a 10 min home cage retention interval. S, sample phase; T, test phase.

12–13 and 18–20-month-old mouse cohorts were tested on a battery of memory tasks assessing the recognition of novel objects (Novel Object Recognition task, what memory), object-in-place associations (Object-in-Place task, what-where memory) or spatial novelty (Object Location task, where memory) (Figures 2A–C). Animals were placed in a 60 cm (L) × 60 cm (W) × 40 cm (H) open-field maze containing an array of objects and contact times with the objects were used to infer novelty detection and thus determine memory function for different attributes of recognition memory.

All tasks consisted of two identical sample phases followed by a test phase. In the test phase, a feature of the object array was changed (i.e., familiar object replaced with novel object, two objects exchanged place, or object moved to a novel location). Before and between each experimental phase, objects and maze were cleaned with 70% ethanol wipes to eliminate odor cues. Positions of the objects within the maze and selection of target objects were counterbalanced among the mice, employing as many different setups as possible. Testing with different tasks was separated by at least a 24 h home cage rest period.

The experiments were recorded with an overhead camera, a Philips DVD-R recorder and a Hitachi television. Contact time with the objects was scored manually with a stopwatch, whereby contact behavior was defined as the animal exploring the object from a distance not greater than 1 cm and facing the object. Passive sitting or grooming in front of an object, attempts to climb an object or chew parts of an object, were not considered explorative behavior and were not scored. To obtain a preference for novelty independent of overall contact time during the test phase and relative to each animal’s individual contact times, a discrimination ratio was calculated as follows: contact time with one target object/(contact time with one target object + contact time with one non-target object). The discrimination ratio ranged from 0 to 1, where ratios above 0.5 indicated a preference for novelty.

### 2.4 Synaptosome extraction

Approximately 4 weeks after completion of the behavioral testing, fresh hippocampus and frontal cortex samples were collected in the morning from Dp1Tyb, Dp(17)3Yey, and Dp(10)2Yey mice that had been used in behavioral experiments and their WT controls. Tissues were immediately snap frozen in liquid nitrogen. Samples were fractionated into purified synaptosome and cytosolic fractions, using Syn-PER reagent (10 μL/1 mg) (ThermoFisher Scientific) as per the manufacturer’s instructions, with protease (1,100) and phosphatase (1,50) inhibitor cocktails (Set III, Merck Millipore). Samples were homogenized and centrifuged (10 min at 1,200 × g, then 30 min at 1,300 × g, at 4°C), the supernatant (cytosolic fraction) was collected and the insoluble pellet (synaptosome fraction) was re-suspended into Syn-PER (1.5 μL/1 mg). The protein concentration of extractions was quantified with a Bicinchoninic Acid Assay (BCA) kit (ThermoFisher Scientific). The synaptosome extraction protocol was validated with immunoblots (Supplementary Figure S1).

### 2.5 Immunoblotting

Expression of glutamate receptors in synaptosome fractions was assessed with immunoblots using standard methods. Twenty microgram of protein was resolved on a 7.5% sodium dodecyl sulfate-polyacrylamide gel and was electrophoresed at 40 mV for 30 min, then 140 mV for 90 min; the proteins in the gel were blotted onto a 0.45 μm nitrocellulose electrode membrane (GE Healthcare Life Sciences). For each mouse line, WT and mutant mice were equally distributed across gels and a WT mouse was randomly selected as internal control for intra-gel variability. Membrane strips were incubated with primary and secondary antibodies, and immunoreactions were detected following incubation in luminol-based enhanced chemiluminescence HRP substrates (SuperSignal West Dura Extended Duration Substrate or Pierce ECL Western Blotting Substrate, ThermoFisher Scientific) with a chemiluminescence machine (G:BOX Chemi XX6, Syngene). Optical density scores of the blots were quantified using NIH ImageJ (1.8.0) and were normalized to internal controls and to the expression of the housekeeping protein β-actin.

### 2.6 Antibodies

Optimal primary antibody dilutions were determined beforehand. For synaptosome preparations: GluN2A (1:1,000, rabbit, Merck Millipore), GluN2B (1:500, rabbit, Merck Millipore), GluN1 (1:500, mouse, BD Biosciences) and pGluN2B(Y1472) (1:750, rabbit, Merck Millipore, AB5403), GluA1 (1:2,500 synaptosome, rabbit, Abcam, ab31232), pGluA1(S845) (1:2,500, rabbit, Abcam, ab3901), GluK5 (1,500, rabbit, Novus Biologicals, NBP1-80270), PSD95 (1,1,000, rabbit, Abcam), β-actin (1,15,000, mouse, Sigma-Aldrich). For cytosol preparations: GluA1 (1,350, rabbit, Abcam, ab31232), DYRK1A (1,500, rabbit, Abnova).

### 2.7 Statistical analyses

Statistical analyses were conducted separately for each mouse line in each experiment with IBM SPSS Statistics 25 and GraphPad Prism 8.4.3. Normal distribution was evaluated with Shapiro–Wilk tests (*p* > 0.05), skewness, kurtosis, histograms, and Q-Q plots, and data that violated the assumptions of parametric statistics were analyzed with analogous non-parametric tests. For all analyses the α-level was set at *p* < 0.05.

In recognition memory tasks, contact times with the objects were analyzed using repeated measures two-way analyses of variance (ANOVAs) with genotype as between-subjects factor (WT vs. mutant) and sample phase (first vs. second) or object (target vs. non-target) as within-subjects factor. Interactions were analyzed with Bonferroni or LSD adjusted tests of simple main effects or Welch’s test in case Levene’s test of homogeneity of variance was violated (*p* < 0.05). Mean discrimination ratios were compared against chance (0.5) with one-sample *t*-tests and group differences analyzed with independent samples Student’s *t*-tests. In the EPM test, scores generated with Ethovision were compared between WT and mutant mice with independent samples Student’s *t*-tests.

For immunoblots, protein density levels were normalized to the means of the WT group to determine changes in protein expression relative to WT levels. Protein expression levels were compared between the groups with independent samples Student’s *t*-tests, separately for each protein of interest. For the Dp(10)2Yey, two-way repeated measures ANOVA were run for each protein of interest, with genotype as between-subjects factor (WT vs. mutant) and age (14 vs. 22 months) as within-subject factor.

## 3 Results

### 3.1 Behavior of Down syndrome mouse models at 8–12 months of age

#### 3.1.1 Dp(17)3Yey mice are hyperactive

**Figure 3:**
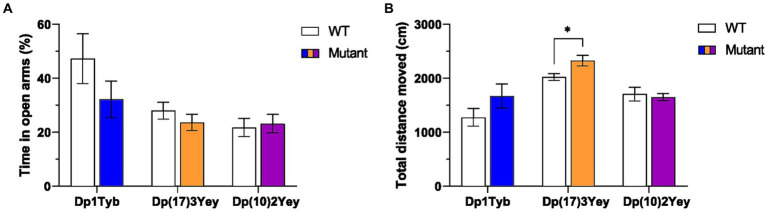
Behavior of Dp1Tyb, Dp(17)3Yey and Dp(10)2Yey male cohorts on the Elevated Plus maze (EPM). **(A)** Mean ± SEM ratio of time spent in open relative to closed arms on the EPM. **(B)** Mean ± SEM total distance traveled on the EPM. Mutant Dp(17)3Yey mice moved a significantly larger distance compared to WT littermates (Student’s *t*-test, *p < 0.05). Dp1Tyb: *n* = 8 WT, 11 Dp1Tyb; Dp(17)3Yey: *n* = 12 WT, 12 Dp(17)3Yey; Dp(10)2Yey: *n* = 12 WT, 12 Dp(10)2Yey.

Since altered anxiety or locomotor activity levels can affect performance in recognition memory tasks, we tested 8–12 month old mice from the three strains that span the Hsa21 region of homology [Dp1Tyb, Dp(17)3Yey, Dp(10)2Yey] using an elevated plus maze (EPM). In all three strains, the ratio of time spent in open versus closed arms of the EPM did not significantly differ between WT and mutant animals, suggesting that none of the strains have altered anxiety levels (Figure 3A; Supplementary Table S1). By contrast, the total distance traveled on the EPM during the trial was significantly higher in Dp(17)3Yey mice relative to WT animals, while no difference was observed in the Dp1Tyb and Dp(10)2Yey strains, implicating the Hsa21-orthologous region on Mmu17 in hyperactivity phenotypes (Figure 3B; Supplementary Table S1). We noted that Dp(17)3Yey mice habituated normally to the EPM (Supplementary Figure S2), suggesting that hyperactivity is not the result of a cognitive defect in encoding the environment.

#### 3.1.2 No difference in baseline object-sampling behavior

To determine the effects of an extra copy of the Hsa21-orthologous regions of Mmu16, Mmu17, and Mmu10 on memory function, we tested 12–13 month old Dp1Tyb, Dp(17)3Yey, and Dp(10)2Yey male mice and their WT littermate controls in three different object-based recognition memory tests (Figures 2A–C). Firstly, contact times during sample phases were analyzed (Table 1) to evaluate whether baseline object exploration differed between WT and mutant animals, especially in light of the hyperactivity phenotype observed in Dp(17)3Yey animals. Contact times were expected to decrease across the two identical sample phases, indicating habituation to the object array. The three strains were first tested on the NOR and the OiP task, in counterbalanced order, and contact times during sample phases were thus averaged across the two tasks.

**Table 1:** Contact times of adult 12–13 month-old Dp1Tyb, Dp(17)3Yey, and Dp(10)2Yey male cohorts during sample phases of recognition memory tasks.

Task	Genotype	Mean contact time	
		S1	S2
NOR & OiP	WT	7.02 (±0.59)	5.04 (±0.46)
	Dp1Tyb	6.37 (±0.60)	4.53 (±0.56)
	WT	13.70 (±1.13)	10.00 (±0.96)
	Dp(17)3Yey	13.94 (±2.00)	9.52 (±1.23)
	WT	12.99 (±1.88)	8.69 (±1.29)
	Dp(10)2Yey	9.64 (±1.01)	8.42 (±1.19)
Loc	WT	5.63 (±0.61)	5.00 (±0.92)
	Dp1Tyb	3.71 (±0.67)	3.57 (±0.62)

All tasks comprised two identical sample phases (S1 and S2) (see Figure 2). The NOR and the OiP tasks comprised a total of three different objects, the Loc task three identical objects. Values are mean (±SEM) contact times with one object. For NOR and OiP task: Dp1Tyb: *n* = 11 WT, 12 Dp1Tyb; Dp(17)3Yey: *n* = 12 WT, 12 Dp(17)3Yey; Dp(10)2Yey: *n* = 12 WT, 12 Dp(10)2Yey. For Loc task: Dp1Tyb: *n* = 11 WT, 11 Dp1Tyb.

In all three strains, contact times did not differ significantly between WT and mutant animals, and contact times decreased significantly across the two sample phases, except for Dp(10)2Yey animals which displayed stable contact times (Supplementary Table S2). Dp1Tyb mice were also tested on the Loc recognition task and again no difference was observed in baseline object exploration (Supplementary Table S2).

#### 3.1.3 Preserved recognition memory for objects in all 3 strains (*what*, NOR task)

To investigate if any of the three Hsa21-orthologous regions of the mouse genome harbor dosage-sensitive genes that affect recognition memory for visual objects, we tested mice from the three strains in the NOR task. In this task, intact memory processes were inferred from higher contact times with novel than familiar objects.

**Figure 4:**
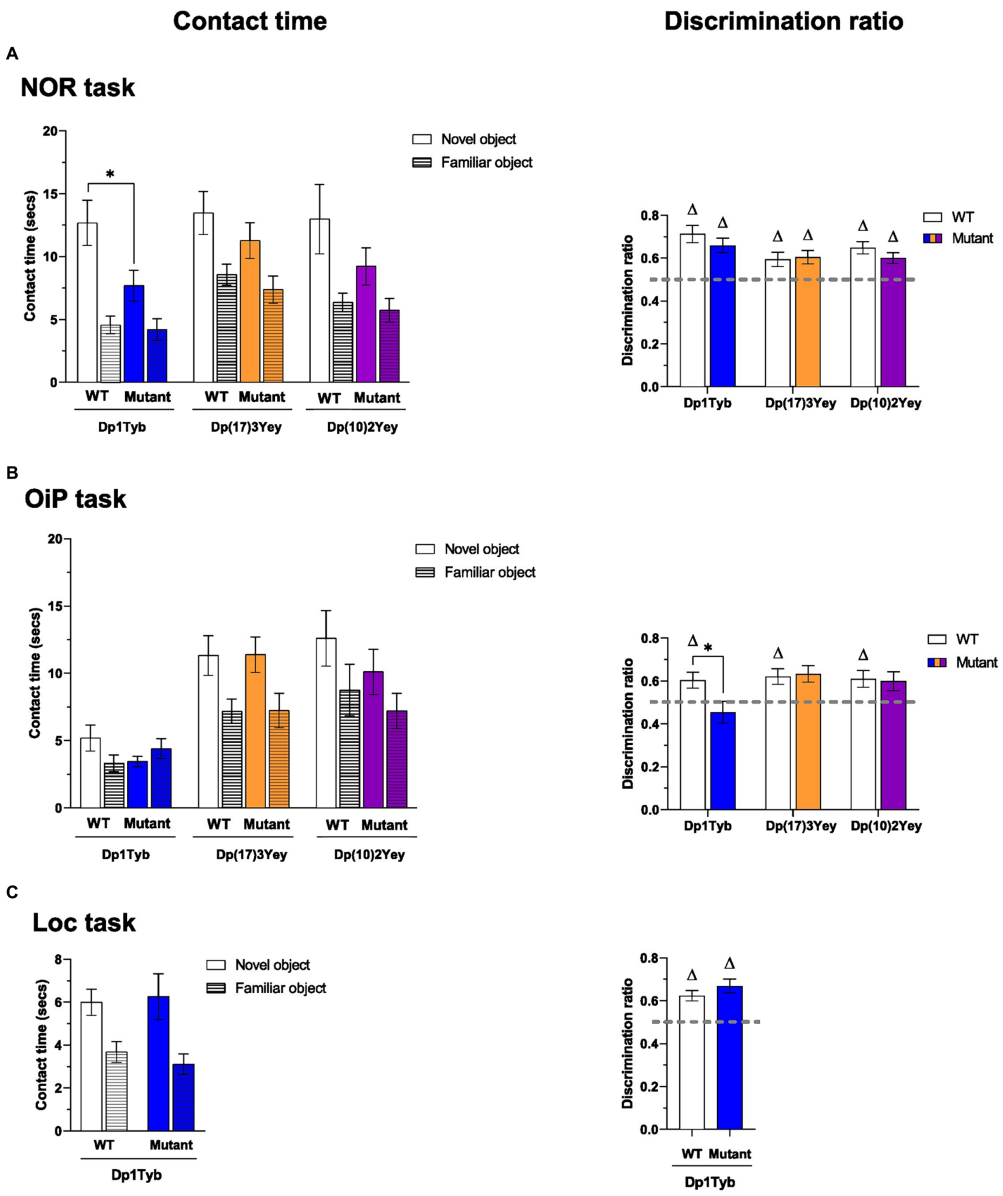
Performance of 12-13-month-old Dp1Tyb, Dp(17)3Yey and Dp(10)2Yey male mice in recognition memory tasks. **(A–C)** Mean ± SEM contact times per single object (left) and discrimination ratios (right) of 12–13 month-old Dp1Tyb, Dp(17)3Yey and Dp(10)2Yey mice and WT controls during the test phase of the recognition memory tasks. In the NOR task **(A)**, the test phase comprised a total of one novel and two familiar objects. In the OiP task **(B)**, the test phase comprised a total of two objects with a novel OiP association and one object with a familiar OiP association. In the Loc task **(C)**, the test comprised a total of one object in a novel location and two identical, stationary objects. Discrimination ratios above 0.5 (gray horizontal lines) indicate above chance preference for novelty (one sample *t*-test, ^∆^*p* < 0.05). Compared to WT littermates, Dp1Tyb mice spent less time with the novel object in the NOR task (two-way ANOVA, *p < 0.05) **(A)** and showed a lower discrimination ratio in the OiP task (Student’s *t*-test, *p < 0.05) **(B)**. For NOR and OiP task: Dp1Tyb: *n* = 11 WT, 12 Dp1Tyb; Dp(17)3Yey: *n* = 12 WT, 12 Dp(17)3Yey; Dp(10)2Yey: *n* = 12 WT, 12 Dp(10)2Yey. For Loc task: Dp1Tyb: *n* = 11 WT, 11 Dp1Tyb.

Both Dp1Tyb and WT controls displayed significantly higher contact times with novel compared to familiar objects but contact times with novel objects were significantly lower in the Dp1Tyb relative to the WT group (Figure 4A; Supplementary Table S3). Next, to assess preference for novelty relative to the animal’s individual contact times, mean discrimination ratios were compared. Despite the observed difference in contact times, mean discrimination ratios of both the WT and the Dp1Tyb mice were significantly above chance and did not differ significantly between each other (Supplementary Table S3). Thus, although novel object contact time was reduced in Dp1Tyb mice, discrimination between novel and familiar objects was preserved.

Both Dp(17)3Yey and Dp(10)2Yey mice and their WT controls displayed significantly higher contact times with novel than familiar objects, to a comparable extent (Figure 4A; Supplementary Table S3). Furthermore, all mean discrimination ratios were significantly above chance and did not differ between WT and mutant mice. Thus, recognition memory for objects was unaltered in Dp(17)3Yey and Dp(10)2Yey mice.

#### 3.1.4 Dp1Tyb mice have impaired recognition memory for object-in-place associations (*what-where*, OiP task)

We further interrogated recognition memory in the three mouse strains by testing associative visuo-spatial memory in the OiP task. Here, intact memory processes were inferred from higher contact times with objects that exchanged locations (i.e., novel OiP associations) compared to stationary objects (i.e., familiar OiP associations).

While overall contact times did not differ between WT and Dp1Tyb animals, only WT mice displayed significantly higher contact times with novel than familiar OiP associations (Figure 4B; Supplementary Table S4). Indeed, the mean discrimination of Dp1Tyb animals did not differ from chance and was significantly lower compared to WT controls. Thus, associative recognition memory for OiP associations was impaired in Dp1Tyb mice.

By contrast, no impairment in OiP memory was observed in Dp(17)3Yey and Dp(10)2Yey mice. Both WT and mutant animals displayed significantly higher contact times with objects that exchanged place than with stationary objects, to a similar extent (Figure 4B; Supplementary Table S4). Furthermore, mean discrimination ratios of both Dp(17)3Yey and Dp(10)2Yey mice were, respectively, significantly above chance and borderline significantly above chance and more critically, did not differ from their WT controls.

#### 3.1.5 Dp1Tyb mice have preserved recognition memory for spatial locations (*where*, Loc task)

To determine whether the OiP memory impairment observed in Dp1Tyb mice was due to a general spatial memory deficit or to a specific deficit in the binding of object and place information, the Dp1Tyb cohort was further tested in the Loc task. This task allowed assessment of recognition memory for spatial locations independently of object information, as three identical objects were used. Intact spatial location memory was inferred from greater contact times with objects moved to a novel, previously vacant location, compared to identical but stationary objects.

Both WT and Dp1Tyb animals displayed significantly higher contact times with objects moved to a novel location, to a similar extent (Figure 4C; Supplementary Table S5). Indeed, mean discrimination ratios of both groups were significantly above chance and did not significantly differ between each other. Thus, despite the OiP memory impairment, Dp1Tyb animals displayed intact recognition memory for spatial locations.

### 3.2 Behavior of Down syndrome mouse models at 18–20 months of age

#### 3.2.1 Dp1Tyb and Dp(10)2Yey mice have reduced baseline object sampling levels

To identify the Hsa21-orthologous regions implicated in age-dependent cognitive decline in DS, we tested the spared memory function of the three mouse strains at 18–20-months in the object-based recognition memory tasks. Since we previously found that memory for OiP associations was impaired in younger Dp1Tyb mice, older Dp1Tyb mice were only tested in the NOR task. To determine whether aging affected baseline object exploration in the three models, contact times during sample phases were analyzed (Table 2; Supplementary Table S6). Dp1Tyb animals displayed significantly reduced contact times compared to WT controls in the first but not second sample phase, and contacts remained stable across the two identical sample phases in the mutant group. Similarly, Dp(10)2Yey mice spent significantly less time sampling the objects compared to their WT controls, but contacts remained stable across the two sample phases in both the WT and Dp(10)2Yey group. By contrast, contact times during sample phases did not differ between WT and Dp(17)3Yey animals, and remained stable across the two sample phases. Dp(10)2Yey mice were also tested on the Loc task, where no difference between the groups was observed during the sample phases.

**Table 2:** Contact times of adult 18–20-month old Dp1Tyb, Dp(17)3Yey, and Dp(10)2Yey male cohorts during sample phases of recognition memory tasks.

Task	Genotype	Mean contact time	
		S1	S2
NOR	WT	11.35 (±1.16)	9.20 (±1.01)
	Dp1Tyb	6.26 (±1.43)	6.76 (±1.13)
	WT	4.98 (±0.60)	4.20 (±0.58)
	Dp(10)2Yey	3.71 (±0.47)	2.97 (±0.28)
NOR & OiP	WT	8.32 (±0.74)	7.40 (±0.94)
	Dp(17)3Yey	9.01 (±0.74)	8.33 (±1.02)
Loc	WT	3.53 (±0.47)	3.37 (±0.41)
	Dp(10)2Yey	3.60 (±0.63)	3.05 (±0.85)

All tasks comprised two identical sample phases (S1 and S2) (see Figure 2). The NOR and the OiP tasks comprised a total of three different objects, the Loc task three identical objects. Values are mean (±SEM) contact times with one object. For all tasks: Dp1Tyb: *n* = 9 WT, 6 Dp1Tyb; Dp(17)3Yey: *n* = 9 WT, 12 Dp(17)3Yey; Dp(10)2Yey: *n* = 9 WT, 11 Dp(10)2Yey.

#### 3.2.2 Older Dp(10)2Yey mice have impaired recognition memory for objects (*what*, NOR task)

**Figure 5:**
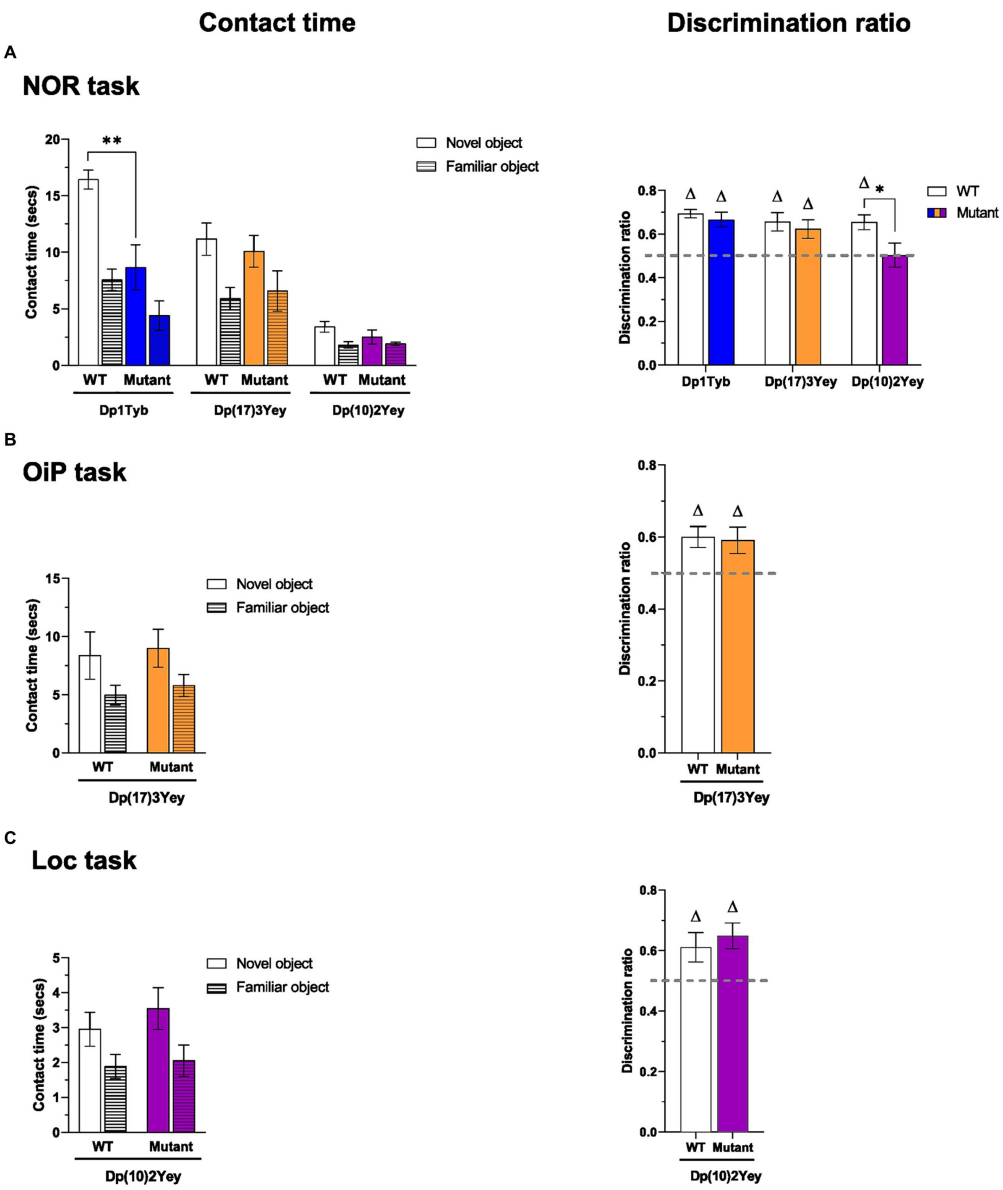
Performance of 18–20-month old Dp1Tyb, Dp(17)3Yey, and Dp(10)2Yey male mice in recognition memory tasks. **(A–C)** Mean ± SEM contact times per single object (left) and discrimination ratios (right) of 18–20-month-old Dp1Tyb, Dp(17)3Yey, and Dp(10)2Yey mice and WT controls during the test phase of the recognition memory tasks as described in Figure 4. Discrimination ratios above 0.5 (gray horizontal lines) indicate above chance preference for novelty (one sample *t*-test, ^∆^*p* < 0.05). Mutant Dp1Tyb mice spent less time with the novel object in the NOR task **(A)** compared to WT littermates (two-way ANOVA, **p < 0.01). Mutant Dp(10)2Yey mice showed a lower discrimination ratio in the NOR task compared to their WT littermates (Student’s *t*-test, *p < 0.05). For NOR and Loc task: Dp1Tyb: *n* = 9 WT, 6 Dp1Tyb; Dp(17)3Yey: *n* = 9 WT, 12 Dp(17)3Yey; Dp(10)2Yey: *n* = 9 WT, 11 Dp(10)2Yey. For OiP task: Dp(17)3Yey: *n* = 8 WT, 12 Dp(17)3Yey.

To investigate if aging affected recognition memory for visual objects in any of the three Hsa21-orthologous models, we tested 18–20-month old mice from the three strains in the NOR task (Figure 5A; Supplementary Table S7). Both aged Dp1Tyb and WT mice displayed significantly higher contact times with novel than familiar objects but contact time with novel objects was significantly lower in the Dp1Tyb compared to the WT group, as had been seen in 12–13 month-old animals. Nevertheless, the mean discrimination ratio of the Dp1Tyb group was significantly above chance and did not differ from the WT group. Thus, novel object contact time is reduced in Dp1Tyb mice but discrimination between novel and familiar objects remains unimpaired even in older animals.

In Dp(10)2Yey animals, aging impaired NOR performance. Although contact times were significantly higher with novel than familiar objects and did not significantly differ between WT and Dp(10)2Yey animals, the mean discrimination ratio analysis revealed that Dp(10)2Yey mice failed to discriminate between novel and familiar objects (Figure 5A; Supplementary Table S7). In contrast to mean contact times, the discrimination ratio takes into account individual differences in the relative time spent with each object type. The mean discrimination ratio of the Dp(10)2Yey group was at chance level and was significantly lower compared to the WT group. Thus, older Dp(10)2Yey mice have impaired novel object recognition memory.

In the Dp(17)3Yey strain, both aged WT and mutant animals displayed significantly higher contact times with novel than familiar objects, to a similar extent (Figure 5A; Supplementary Table S7). Accordingly, mean discrimination ratios of both groups were significantly above chance and did not differ between each other. Thus, novel object recognition memory is preserved even in older Dp(17)3Yey mice.

#### 3.2.3 Older Dp(17)3Yey mice have unimpaired recognition memory for object-in-place associations (*what-where*, OiP task)

To further interrogate cognitive function in older Dp(17)3Yey mice, we tested visuo-spatial memory function in the OiP task. Once again, both WT and Dp(17)3Yey animals showed significantly higher contact times with objects that exchanged place relative to objects that remained in the same spatial location, to a similar extent (Figure 5B; Supplementary Table S8). Mean discrimination ratios of both groups were significantly above chance and did not significantly differ between them. Aging, therefore, did not impair associative recognition memory function in Dp(17)3Yey mutant mice.

Since we found that memory for OiP associations was impaired already in younger Dp1Tyb mice, this group was not re-investigated in the OiP task at the older age. Similarly, since older Dp(10)2Yey animals presented a deficit in the NOR task and memory for OiP associations cannot be assessed independently of memory for objects, this group was also not investigated in the OiP task.

#### 3.2.4 Older Dp(10)2Yey mice have preserved recognition memory for spatial locations (*where*, Loc task)

To assess the behavioral specificity of the NOR deficit observed in older Dp(10)2Yey mutant mice, this strain was further tested in the Loc task, which investigates spatial novelty detection independently of memory for object identity. Both WT and Dp(10)2Yey mice showed significantly higher contact times with objects moved to a previously vacant location relative to an object that remained in the same spatial location (Figure 5C; Supplementary Table S9). Mean discrimination ratios of both groups were significantly above chance and did not differ significantly between each other. Thus, aging in the Dp(10)2Yey did not disrupt recognition memory overall but specifically affected novel object detection while preserving recognition memory for spatial locations.

### 3.3 Hippocampal immunoblots of Down syndrome mouse models

#### 3.3.1 Unaltered gross hippocampal dissection volume

To determine whether the observed memory impairments occurred in conjunction with altered expression of glutamate receptors known to subserve recognition memory function, we assessed hippocampal levels of AMPA, NMDA, and KA subunits by immunoblotting HPC synaptosome extracts from Dp1Tyb, Dp(17)3Yey, and Dp(10)2Yey cohorts previously used in behavioral experiments. For the Dp1Tyb and Dp(17)3Yey strains, samples were collected at 21 months of age. Given the age-dependent memory deficit of Dp(10)2Yey mutant mice, samples were collected and compared between 14 and 22-month-old groups. Even though body weight was significantly reduced in Dp1Tyb mice compared to WT littermates (Supplementary Table S10), we noted that hippocampal dissection weight did not significantly differ between WT and mutant animals in the three strains, suggesting that gross hippocampal volume was not altered by increased dosage of any of the Hsa21-orthologous regions (Supplementary Table S11) and in Dp1Tyb with more sensitive regional volumetric analyses.

#### 3.3.2 Increased GluA1 overexpression in Dp1Tyb HPC synaptosomes

**Figure 6:**
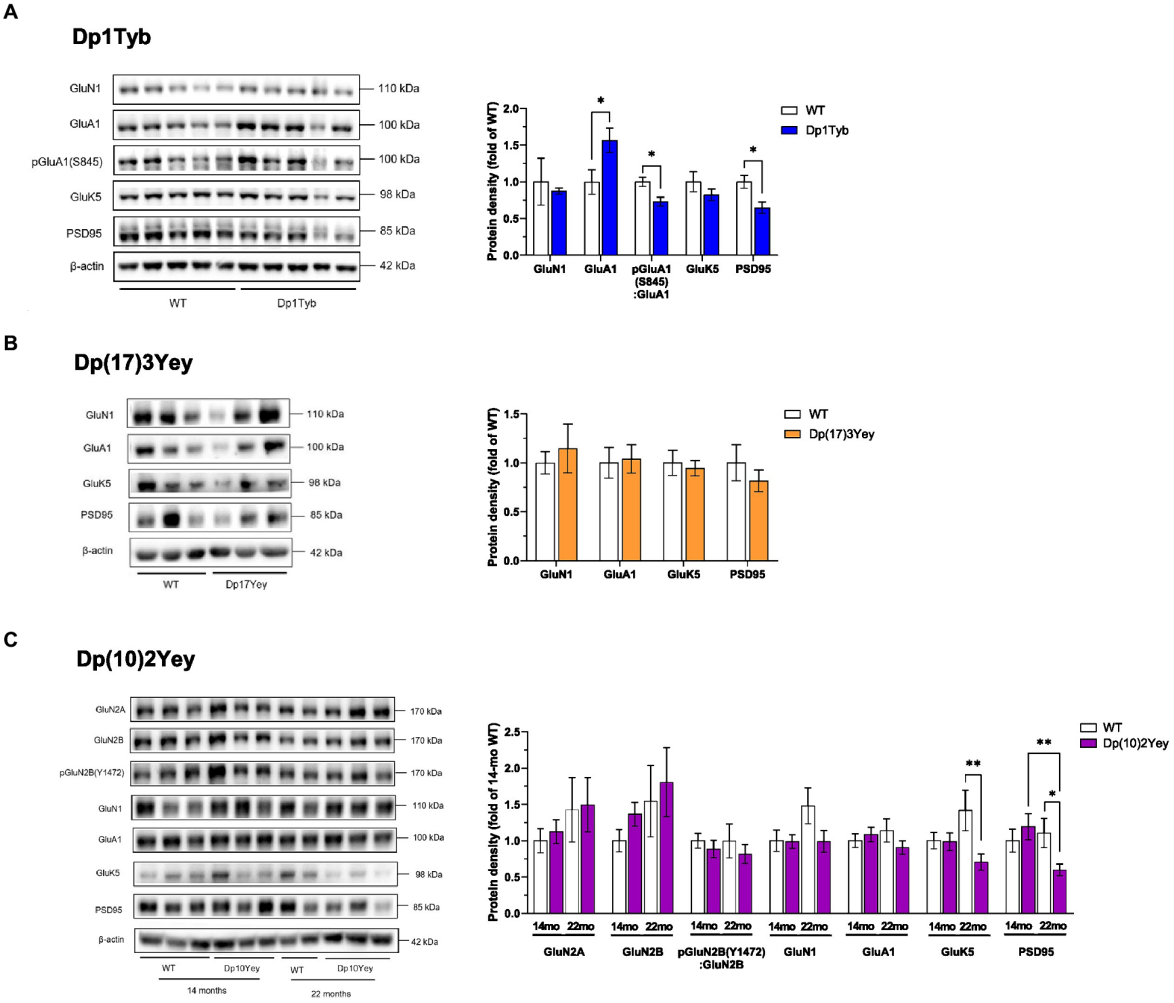
Altered abundance of glutamate receptor subunits in HPC synaptosomes from Dp1Tyb, Dp(17)3Yey, and Dp(10)2Yey male mice. **(A–C)** HPC synaptosomes from the indicated mouse strains were analyzed by immunoblotting for glutamate receptor subunits and PSD95. Example immunoblots are shown on the left and mean ± SEM protein abundance on the right, normalized to β-actin (or to GluA1 or GluN2B in case of phosphorylation levels of the respective receptor) and then to the mean signal in WT mice. Immunoblots show analysis of HPC synaptosome extracts from 2 to 5 mice of each genotype. Compared to WT littermates, mutant Dp1Tyb mice displayed lower hippocampal expression of GluA1, pGluA1(S845), and PSD95 (Student’s *t*-test, *p < 0.05) **(A)**. Mutant Dp(10)2Yey mice displayed an age-dependent decrease in hippocampal expression of GluK5 and PSD95 compared to WT littermates (two-way ANOVA, *p < 0.05, **p < 0.001) **(C)**. The decrease in GluK5 expression was not driven by age-dependent changes in the WT group as this comparison was not statistically significant (*p* > 0.05) **(C)**. 21-month-old Dp1Tyb: *n* = 6 WT, 5 Dp1Tyb; 21-month-old Dp(17)3Yey: *n* = 9 WT, 12 Dp(17)3Yey; 14-month-old Dp(10)2Yey: *n* = 9 WT, 11 Dp(10)2Yey; 22-month-old Dp(10)2Yey: *n* = 6 WT, 10 Dp(10)2Yey.

In the Dp1Tyb strain, immunoblot analysis revealed that levels of the GluN1 NMDA- and the GluK5 KA-receptor subunits in the HPC were similar to WT controls at 21 months of age (Figure 6A; Supplementary Table S12). By contrast, Dp1Tyb animals displayed a significant upregulation of the GluA1 AMPA receptor subunit compared to WT levels. Interestingly, the GluA1 overexpression was not accompanied by an increase in GluA1 phosphorylation as GluA1 S845 phosphorylation levels relative to total GluA1 were significantly decreased. Hippocampal synaptosomes from Dp1Tyb animals also displayed a significant reduction in the expression of the major post-synaptic scaffolding protein PSD95. These changes were not present in frontal cortex synaptosomes (Supplementary Figure S3; Supplementary Table S13).

**Figure 7:**
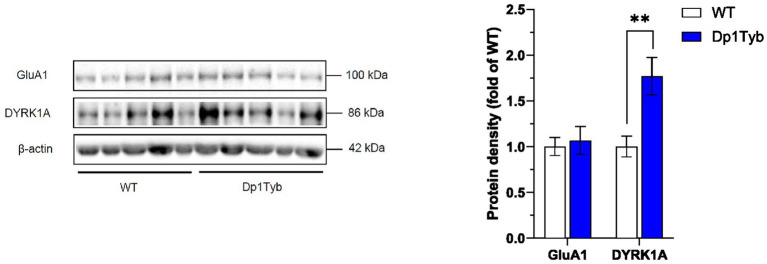
Increased DYRK1A abundance in HPC cytosol from Dp1Tyb male mice. HPC cytosolic extracts from Dp1Tyb and WT mice were analyzed by immunoblotting for GluA1 and DYRK1A. Example immunoblot is shown on the left and mean ± SEM protein abundance on the right, normalized to β-actin and then to the mean signal in WT mice. Immunoblot shows analysis of HPC extracts from 5 mice of each genotype. Mutant Dp1Tyb mice displayed higher DYRK1A expression in hippocampal cytosol compared to WT littermates (Student’s *t*-test, **p < 0.01). 21-month-old Dp1Tyb: *n* = 6 WT, 5 Dp1Tyb.

To determine whether the observed hippocampal GluA1 overexpression was restricted to the synaptic compartment or was accompanied by a corresponding increase in the intracellular GluA1 receptor pool, GluA1 levels were also assessed in cytosol fractions of the HPC. Unlike synaptosome extracts, GluA1 expression in the cytosol did not differ between WT and Dp1Tyb animals (Figure 7; Supplementary Table S12).

Next, to investigate a possible mechanism related to GluA1 accumulation, we assessed levels of the DYRK1A serine/threonine protein kinase, encoded by the *Dyrk1a gene* which is triplicated in the Dp1Tyb strain. DYRK1A has been previously implicated in cognitive dysfunction and in receptor trafficking (Grau et al., 2014; Souchet et al., 2014) and is primarily located in the cytosol (Supplementary Figure S4; Supplementary Table S12). In line with the increased gene dosage, levels of DYRK1A were significantly upregulated in the HPC cytosol of Dp1Tyb mice compared to WT mice (Figure 7; Supplementary Table S12).

#### 3.3.3 Unchanged glutamate receptor expression in Dp(17)3Yey HPC synaptosomes

In the Dp(17)3Yey strain, at 21 months of age, immunoblotting showed unaltered expression levels of glutamate receptor subunits and of PSD95 (Figure 6B; Supplementary Table S12).

#### 3.3.4 Reduced levels of GluK5 in HPC synaptosomes from older Dp(10)2Yey mice

Since the Dp(10)2Yey strain displayed age-dependent memory deficits, hippocampal protein expression was assessed at 14 months and 22 months of age. Immunoblotting showed that there was no change in expression of AMPA or NMDAR subunits in HPC synaptosomes at either age, with samples from WT and Dp(10)2Yey mice displaying comparable levels of GluA1, GluN1, GluN2A, and pGluN2B(Y1472) (Figure 6C; Supplementary Table S14). In contrast, while no change was seen at 14 months of age in the expression of GluK5 and PSD95, 22-month old Dp(10)2Yey mice had significantly decreased hippocampal levels of both GluK5 and PSD95 compared to their WT littermates (Figure 6C; Supplementary Table S14). These changes were not present in frontal cortex synaptosomes (Supplementary Figure S3B; Supplementary Table S13).

## 4 Discussion

DS is a complex disorder caused by trisomy of Hsa21. We examined the separate contribution of the Mmu16, Mmu17, and Mmu10 conserved regions to recognition memory function in adult and aged mouse models of Hsa21 partial trisomy. The selection of recognition tasks was designed to interrogate different attributes of recognition memory. The results reveal different patterns of performance between mutants and across different ages. At 12–13 months of age, Dp1Tyb, Dp(17)3Yey, and Dp(10)2Yey mice were all able to recognize novel objects among familiar objects (*what memory*). Adult Dp(17)3Yey and Dp(10)2Yey mice also displayed intact memory for OiP associations (*what-where memory*), while Dp1Tyb mice were impaired. Interestingly, this impairment was due to a specific deficit in the binding of object and place information, as adult Dp1Tyb mice were able to identify new spatial locations (*where memory*). At 18–20 months of age, Dp1Tyb and Dp(17)3Yey mice still showed preserved memory for object novelty (*what*) while Dp(10)2Yey mice were no longer able to recognize novel objects at this age. Memory deficits were observed in conjunction with altered glutamate receptor expression in the HPC.

Previous research has reported impaired NOR performance in the very similar Dp(16)1Yey strain following a 24-h delay between sample and test trials (Souchet et al., 2019). Unlike the current study, however, NOR performance with shorter delays was not evaluated. Here, Dp1Tyb mutant mice successfully discriminated between novel and familiar objects after a 10-min delay but contact times with novel objects were significantly reduced at this delay. It can be hypothesized that an extra copy of the Hsa21-orthologous region of Mmu16 may affect longer term object memory while sparing shorter term object memory.

In our study we also observed that adult Dp1Tyb mice displayed a selective impairment in the retention of OiP associations, while preserving object location memory. The binding of item- and place-information has been repeatedly demonstrated to depend on the HPC (Eichenbaum et al., 2007; Warburton and Brown, 2015). Lesions of the HPC impair performance in the OiP task but not NOR performance, the latter appearing more sensitive to disruption of the perirhinal cortex (Barker et al., 2007; Barker and Warburton, 2011). The pattern of behavioral deficits reported in our study is therefore consistent with Mmu16 segmental trisomy disrupting hippocampal function. Indeed, previous research on Dp(16)1Yey mice reported impairments in HPC-dependent memory tasks, such as the Morris Water Maze, and in the induction of hippocampal LTP (Yu et al., 2010a,b; Aziz et al., 2018). Evidence using *in vivo* electrophysiological recordings in Dp1Tyb animals has also found altered hippocampal-prefrontal cortex dynamics during a T-maze spontaneous alternation task (Chang et al., 2020).

In agreement with the behavioral evidence suggesting hippocampal dysfunction in Dp1Tyb mice, we found altered protein expression in this structure, with hippocampal GluA1 surface levels upregulated by ~1.5-fold and GluA1 S845 phosphorylation levels decreased by ~30%. Hippocampal AMPAR activity is a requirement for normal performance on the OiP task (Barker and Warburton, 2015). In DS, AMPAR levels are generally reduced but increased AMPAR expression has also been reported, especially in older subjects (Arai et al., 1996; Siarey et al., 2006). Importantly, the observed GluA1 upregulation selectively affected synaptosome compartments, as GluA1 levels were unaltered in the cytosol fraction, suggesting aberrant AMPAR trafficking at the synapse. In Dp1Tyb mice, hippocampal DYRK1A levels were upregulated by ~1.7-fold, similarly to that seen in human DS brain samples and as would be expected for a gene on Hsa21 that is present in 3 copies in both DS and the Dp1Tyb mouse strain (Dowjat et al., 2007). DYRK1A activity has been implicated in the phosphorylation events underlying glutamate receptor trafficking and surface expression (Grau et al., 2014), and transgenic mice overexpressing DYRK1A display altered glutamate receptor levels and memory deficits (Souchet et al., 2014). The S845 site is a regulatory site for AMPAR cycling, whereby dephosphorylation and phosphorylation of S845 have been, respectively, associated with synaptic removal and insertion of GluA1 subunits, and is also relevant for synaptic plasticity mechanisms and spatial memory performance (Lee et al., 2000, 2003; Kim and Ziff, 2014). It can be hypothesized that reduction in S845 phosphorylation in the Dp1Tyb may indicate that surplus GluA1 subunits were potentially targeted for endocytosis. Additionally, dephosphorylation of S845 is mediated by calcineurin, whose activity is indirectly modulated by DYRK1A (Jung et al., 2011; Kim and Ziff, 2014).

Although an increase in synaptic AMPARs is known to potentiate the synapse (Citri and Malenka, 2008), AMPAR upregulation in the context of prolonged, non-dynamic trafficking may impair the activity-dependent modulation of synaptic strength through AMPAR redistribution, with AMPAR overexpression preventing LTP (Whitlock et al., 2006).

We also noted that HPC synaptosomes of Dp1Tyb animals displayed a ~35% downregulation in PSD95 levels. PSD95 is a core structural protein present in all dendritic spines and reduced PSD95 levels are likely to indicate postsynaptic degeneration (Chen et al., 2011; Shao et al., 2011). Although we found no behavioral evidence of age-dependent cognitive decline in the Dp1Tyb strain, we hypothesize that the metabolic products of proteins encoded by key genes on Mmu16, such as *App*, a gene implicated in the development of Aβ pathology and AD (Cannavo et al., 2020), may promote postsynaptic density loss and neurodegeneration. This seems possible especially in light of the fact that Aβ oligomers preferentially target PSD95-sites (Lacor et al., 2004) and that increased cortical Aβ levels have been reported in Dp(16)1Yey animals (Sawa et al., 2021).

Dp(17)3Yey mice displayed intact performance in all recognition memory tasks, even at advanced age. Immunoblots of the HPC synaptosome also found no alterations in glutamate receptor expression. Together, these findings suggest that the Mmu17 trisomy did not affect medial temporal lobe structures underlying recognition memory function. In line with this interpretation, previous studies found that the Dp(17)3Yey strain was not associated with aberrant hippocampal electrophysiology or memory impairments (Yu et al., 2010a,b; Chang et al., 2020). Nevertheless, *in vitro* recordings showed that hippocampal LTP induction was significantly increased in Dp(17)3Yey mice (Yu et al., 2010a,b).

Although we observed no deficits in object-based recognition memory tasks in Dp(17)3Yey mice, they showed increased locomotor activity in the EPM test, suggesting that increased dosage of Hsa21-orthologous genes on Mmu17 may contribute to activity disorders commonly observed in DS (Ekstein et al., 2011). Interestingly, evidence from the Dp1Yah model, which contains an additional copy of only a part of the Hsa21-orthologous region of Mmu17, reported the opposite pattern of results, with Dp1Yah mice displaying impaired memory for novel objects and normal locomotor activity levels (Pereira et al., 2009; Marechal et al., 2019). Genetic differences between the two models may thus account for the opposite behavioral phenotypes observed.

Finally, we showed an age-dependent object recognition memory deficit in Dp(10)2Yey mice, along with age-dependent changes in synaptic protein expression. We conclude that, while increased dosage of the Hsa21-orthologous region of Mmu10 did not interfere with activity of the medial temporal lobe subserving recognition memory, in conjunction with aging it disrupted object novelty detection processes. Interestingly, previous evidence suggests that other aspects of memory function are impaired in Dp(10)2Yey mice independently of age, with mutants displaying a deficit on T-maze alternation at 3 months of age (Chang et al., 2020).

Hippocampal levels of the GluK5 kainate receptor subunit and of PSD95 in Dp(10)2Yey mice did not differ from WT levels at 14 months of age but were downregulated by ~50% at 22 months of age. Previous research has shown that antagonism of GluK5 in the perirhinal cortex impairs NOR performance (Barker et al., 2006) and that kainate receptors, specifically the GluK5 subunit, play a key role in synaptic plasticity mechanisms (Carta et al., 2013; Petrovic et al., 2017). We hypothesize that kainate receptor expression may therefore be altered with age in the perirhinal cortex, as noted in the HPC, underpinning the age-related object novelty decline in Dp(10)2Yey mice.

Additionally, GluK5 downregulation might be directly related to downregulation of PSD95. PSD95 binds to GluK5, anchoring kainate receptors to the postsynaptic density. Unlike other glutamate receptor subunits capable of binding to other postsynaptic density scaffold proteins, GluK5 only binds tightly to PSD95 (Mehta et al., 2001; Won et al., 2017).

PSD95 downregulation in aged but not younger Dp(10)2Yey mice strongly indicates that the Mmu10 trisomy gradually promotes postsynaptic degeneration. Age-dependent postsynaptic loss and cognitive decline may be related to over-dosage of S100B calcium-binding protein B (*S100b*). *In vitro* and *in vivo* evidence reports that S100B may act as a pro-inflammatory cytokine in response to Aβ aggregation and to upregulate APP expression in a self-propagating cycle exacerbating Aβ pathology (Wilcock and Griffin, 2013). In DS, S100B expression is already elevated in fetuses and progressively increases with age, and cortical S100B levels positively correlate with Aβ deposition levels (Griffin et al., 1989). Transgenic mice overexpressing S100B display age-dependent memory deficits and dendritic spine loss (Whitaker-Azmitia et al., 1997). In the Dp(10)2Yey strain, trisomic for *S100b* but not *App*, both S100B and APP hippocampal levels were found to be elevated in 7-9-month-old male mice (Block et al., 2015). Future research should investigate the possibility that, in Dp(10)2Yey mice, over-dosage of *S100b* may progressively lead to Aβ accumulation, neurodegeneration and cognitive decline. Indeed, Aβ deposition in mice is enhanced by several Hsa21-orthologous genes other than *App* (Wiseman et al., 2018; Tosh et al., 2021).

In conclusion, we report that three copies of distinct Hsa21-orthologous regions in male mice have a differential impact on recognition memory function and the synaptic processes that underpin it. It is important to note that our study was restricted to male mice and there is evidence for difference in protein expression levels, skeletal abnormalities and in learning between the male and female DS model mice (Block et al., 2015; Thomas et al., 2020; Ahmed et al., 2021). Further work is therefore required to determine whether there are sex-specific differences in recognition memory and synaptic function in DS mice that interacts with age. Nevertheless, our results suggest that pharmacological modulation of glutamate receptor activity could be investigated in future studies as a treatment to rescue memory deficits and delay cognitive decline.

## Data availability statement

The original contributions presented in the study are included in the article/Supplementary material, further inquiries can be directed to the corresponding authors.

## Ethics statement

The animal study was approved by the UK Home Office in accordance with the United Kingdom Animal (Scientific Procedures) Act 1986. The study was conducted in accordance with the local legislation and institutional requirements.

## Author contributions

TC: Conceptualization, Data curation, Formal analysis, Investigation, Methodology, Visualization, Writing – original draft, Writing – review & editing. EK: Conceptualization, Methodology, Resources, Supervision, Writing – review & editing. DG: Resources, Methodology, Writing – review & editing. EL-E: Resources, Methodology, Writing – review & editing. EF: Conceptualization, Funding acquisition, Resources, Writing – review & editing. VT: Conceptualization, Funding acquisition, Resources, Writing – review & editing. MG: Conceptualization, Funding acquisition, Methodology, Project administration, Resources, Supervision, Writing – original draft, Writing – review & editing.
