# Supplementary material for: Dissecting the contribution of human chromosome 21 syntenic regions to recognition memory processes in adult and aged mouse models of Down syndrome
Source: Front Behav Neurosci. 2024 Jul 10;18:1428146. doi: 10.3389/fnbeh.2024.1428146 (PMC11266108; doi:10.3389/fnbeh.2024.1428146)
Supplement: Supplementary file 1 [file Data_Sheet_1.docx]

# Supplementary figures


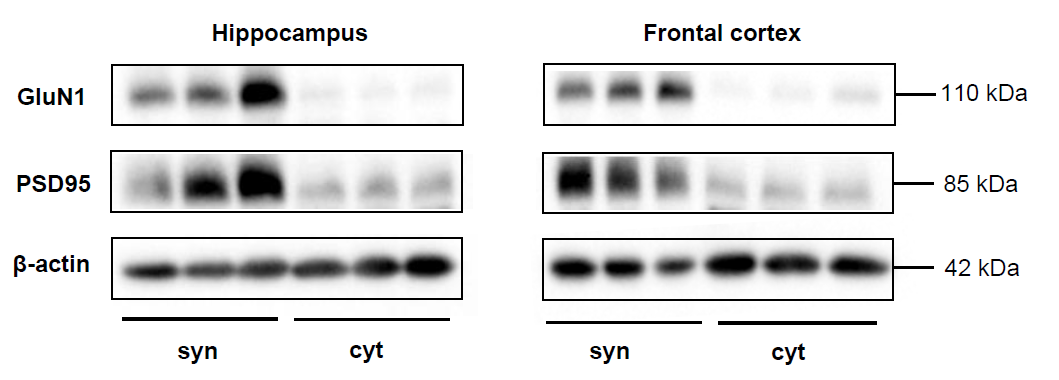


**Figure S1. Immunoblots validating the synaptosome extraction protocol.**

Immunoblots of synaptosome (syn) and cytosolic (cyt) fractions from the hippocampus and frontal cortex of 16-month-old WT mice, showing higher levels of synaptic markers (GluN1 and PSD95) in synaptosome compared to cytosolic fractions, with equivalent β-actin levels. n = 3.

**Figure S2. Dp(17)3Yey male mice habituate to the Elevated plus maze.**

Mean (±SEM) distance travelled per minute by Dp(17)3Yey and WT control mice on the Elevated plus maze. Although Dp(17)3Yey mice travelled a significantly larger distance compared to WT littermates (Figure 3), locomotor activity decreased significantly over time in both lines (two-way ANOVA, genotype: p<0.05, time: p<0.001, genotype x time: p>0.05). n = 12 WT, 12 Dp(17)3Yey.

**Figure S3. Abundance of GluA1, GluK5 and PSD95 in frontal cortex synaptosomes from Dp1Tyb and Dp(10)2Yey male mice.**

(**A, B**) Frontal cortex synaptosomes from the indicated mouse strains were analyzed by immunoblotting for glutamate receptor subunits and PSD95. Example immunoblots are shown on the left and mean±SEM protein abundance on the right, normalized to β-actin and then to the mean signal in WT mice. Immunoblots show analysis of frontal cortex synaptosome extracts from 4-5 mice of each genotype. No significant differences were observed in either of the two mouse lines (Student’s t-test, p>0.05). 21-month-old Dp1Tyb: n= 6 WT, 5 Dp1Tyb; 22-month-old Dp(10)2Yey: n= 5 WT, 11 Dp(10)2Yey.


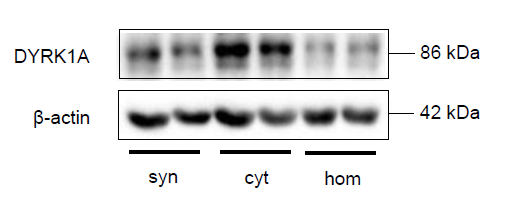

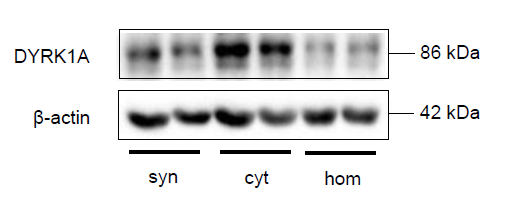


**Figure S4. Immunoblots showing subcellular location of DYRK1A.**

Immunoblot of synaptosome (syn) and cytosol (cyt) extracts of HPC from 16-month-old WT mice. DYRK1A is more abundant in the cytosol relative to synaptosomes.

# Supplementary tables

| **Task** | **Strain** | **Sample size** | **Measure** | **Statistical test** | **Test value** | **p value** |
| --- | --- | --- | --- | --- | --- | --- |
|  |  |  |  |  |  |  |
| EPM | Dp1Tyb | WT = 8 Mut = 11 | Time in open vs closed arms [%] | Student’s t-test | t_(17)_=1.346 | p=0.196 |
|  |  |  | Distance travelled [cm] | Student’s t-test | t_(17)_=1.330 | p=0.201 |
|  |  |  |  |  |  |  |
|  | Dp(17)3Yey | WT = 12 Mut = 12 | Time in open vs closed arms [%] | Student’s t-test | t_(22)_=1.009 | p=0.324 |
|  |  |  | Distance travelled [cm] | Student’s t-test | t_(22)_=2.593 | **p=0.017** |
|  |  |  |  |  |  |  |
|  | Dp(10)2Yey | WT = 12 Mut = 12 | Time in open vs closed arms [%] | Student’s t-test | t_(22)_=0.306 | p=0.762 |
|  |  |  | Distance travelled [cm] | Student’s t-test | t_(16)_=0.388 | p=0.703 |

**Table S1. Statistical comparison of Dp1Tyb, Dp(17)3Yey and Dp(10)2Yey cohorts and their WT littermates on the Elevated Plus Maze (EPM).**Significant results (p<0.05) highlighted in bold.

| **Task** | **Strain** | **Sample size** | **Measure** | **Statistical test** | **Factor** | **Test value** | **p value** |
| --- | --- | --- | --- | --- | --- | --- | --- |
|  |  |  |  |  |  |  |  |
| NOR & OiP at 12-13 months (sample phases) | Dp1Tyb | WT = 11 Mut = 12 | contact time with objects in 2 identical sample phases [sec] | ANOVA | genotype | F_(1,21)_=0.593 | p=0.450 |
|  |  |  |  |  | sample phase | F_(1,21)_=58.12 | **p<0.001** |
|  |  |  |  |  | genotype x sample phase | F_(1,21)_=0.070 | p=0.795 |
|  |  |  |  |  |  |  |  |
|  | Dp(17)3Yey | WT = 12 Mut = 12 | contact time with objects in 2 identical sample phases [sec] | ANOVA | genotype | F_(1,22)_=0.004 | p=0.947 |
|  |  |  |  |  | sample phase | F_(1,22)_=34.16 | **p<0.001** |
|  |  |  |  |  | genotype x sample phase | F_(1,22)_=0.267 | p=0.611 |
|  |  |  |  |  |  |  |  |
|  | Dp(10)2Yey | WT = 12 Mut = 12 | contact time with objects in 2 identical sample phases [sec] | ANOVA | genotype | F_(1,22)_=0.980 | p=0.333 |
|  |  |  |  |  | sample phase | F_(1,22)_=15.47 | **p<0.001** |
|  |  |  |  |  | genotype x sample phase | F_(1,22)_=4.816 | **p=0.039** |
|  |  |  |  |  | simple main effects of genotype | S1: F_(1,22)_=1.711  S2: F_(1,22)_=0.137 | S1: p=0.188  S2: p>0.999 |
|  |  |  |  |  | simple main effects of sample phase | WT: F_(1,22)_=4.333  mut: F_(1,22)_=1.229 | **WT: p<0.001**  Mut: p=0.464 |
|  |  |  |  |  |  |  |  |
| Loc at 12-13 months (sample phases) | Dp1Tyb | WT = 11 Mut = 11 | contact time with objects in 2 identical sample phases [sec] | ANOVA | genotype | F_(1,20)_=3.548 | p=0.074 |
|  |  |  |  |  | sample phase | F_(1,20)_=0.658 | p=0.427 |
|  |  |  |  |  | genotype x sample phase | F_(1,20)_=0.264 | p=0.613 |

**Table S2. Statistical comparison of 12-13-month-old Dp1Tyb, Dp(17)3Yey and Dp(10)2Yey cohorts and their WT littermates during sample phases of recognition memory tasks.** Significant results (p<0.05) highlighted in bold. Mut = Mutant; WT = Wildtype.

| **Task** | **Strain** | **Sample size** | **Measure** | **Statistical test** | **Factor** | **test value** | **p value** |
| --- | --- | --- | --- | --- | --- | --- | --- |
|  |  |  |  |  |  |  |  |
| NOR at 12-13 months (test phase) | Dp1Tyb | WT = 11 Mut = 12 | contact time with novel vs. familiar objects [sec] | ANOVA | genotype | F_(1,21)_=3.307 | p=0.083 |
|  |  |  |  |  | object | F_(1,21)_=44.019 | **p<0.001** |
|  |  |  |  |  | genotype x object | F_(1,21)_=7.028 | **p=0.015** |
|  |  |  |  |  | simple main effects of genotype | nov: F_(1,21)_=5.432  fam: F_(1,21)_=0.103 | nov: **p=0.030**  fam: p=0.751 |
|  |  |  |  |  | simple main effects of object | WT: F_(1,21)_=41.316  Mut: F_(1,21)_=8.295 | WT: **p<0.001**  Mut: **p=0.009** |
|  |  |  | mean discrimination ratio vs. chance (0.5) | one sample t-test |  | WT: t_(10)_=5.253  Mut: t_(11)_=4.697 | WT: **p<0.001**  Mut: **p<0.001** |
|  |  |  | mean discrimination ratio | Student’s t-test |  | t_(21)_=1.050 | p=0.306 |
|  |  |  |  |  |  |  |  |
|  | Dp(17)3Yey | WT = 12 Mut = 12 | contact time with novel vs. familiar objects [sec] | ANOVA | genotype | F_(1,22)_=1.095 | p=0.307 |
|  |  |  |  |  | object | F_(1,22)_=23.881 | **p<0.001** |
|  |  |  |  |  | genotype x object | F_(1,22)_=0.310 | p=0.584 |
|  |  |  | mean discrimination ratio vs. chance (0.5) | one sample t-test |  | WT: t_(11)_=2.876  Mut: t_(11)_=3.325 | WT: **p=0.015**  Mut: **p=0.007** |
|  |  |  | mean discrimination ratio | Student’s t-test |  | t_(22)_=0.216 | p=0.831 |
|  |  |  |  |  |  |  |  |
|  | Dp(10)2Yey | WT = 12 Mut = 12 | contact time with novel vs. familiar objects [sec] | ANOVA | genotype | F_(1,22)_=1.301 | p=0.266 |
|  |  |  |  |  | object | F_(1,22)_=13.077 | **p=0.002** |
|  |  |  |  |  | genotype x object | F_(1,22)_=1.248 | p=0.276 |
|  |  |  | mean discrimination ratio vs. chance (0.5) | one sample t-test |  | WT: t_(11)_=5.269  Mut: t_(11)_=3.904 | WT: **p<0.001**  Mut: **p=0.002** |
|  |  |  | mean discrimination | Student’s t-test |  | t_(22)_=1.273 | p=0.216 |

**Table S3. Statistical comparison of 12-13-month-old Dp1Tyb, Dp(17)3Yey and Dp(10)2Yey cohorts and their WT littermates during the test phase of the NOR task.** Significant results (p<0.05) highlighted in bold. Mut = Mutant; WT = Wildtype.

| **Task** | **Strain** | **Sample size** | **Measure** | **Statistical test** | **Factor** | **test value** | **p value** |
| --- | --- | --- | --- | --- | --- | --- | --- |
|  |  |  |  |  |  |  |  |
| OiP task at 12-13 months (test phase) | Dp1Tyb | WT = 11 Mut = 12 | contact time with novel vs. familiar OiP associations [sec] | ANOVA | genotype | F_(1,21)_=0.169 | p=0.685 |
|  |  |  |  |  | object | F_(1,21)_=0.653 | p=0.428 |
|  |  |  |  |  | genotype x object | F_(1,21)_=6.212 | **p=0.021** |
|  |  |  |  |  | simple main effects of object | WT: F_(1,21)_=5.219 Mut: F_(1,21)_=1.483 | WT: **p=0.033** Mut: p=0.237 |
|  |  |  |  |  | simple main effects of genotype | nov: F_(1,21)_=3.032 fam: F_(1,21)_=1.282 | nov: p=0.096 fam: p=0.270 |
|  |  |  | mean discrimination ratio vs. chance (0.5) | one sample t-test |  | WT: t_(10)_=2.810  Mut: t_(11)_=0.894 | WT: **p=0.018** Mut: p=0.390 |
|  |  |  | mean discrimination | Student’s t-test |  | t_(21)_=2.337 | **p=0.029** |
|  |  |  |  |  |  |  |  |
|  | Dp(17)3Yey | WT = 12 Mut = 12 | contact time with novel vs. familiar OiP associations [sec] | ANOVA | genotype | F_(1,22)_=0.001 | p=0.971 |
|  |  |  |  |  | object | F_(1,22)_=19.418 | **p<0.001** |
|  |  |  |  |  | genotype x object | F_(1,22)_<0.001 | p=0.993 |
|  |  |  | mean discrimination ratio vs. chance (0.5) | one sample t-test |  | WT: t_(11)_=3.345 Mut: t_(11)_=3.452 | WT: **p=0.007**  Mut: **p=0.005** |
|  |  |  | mean discrimination | Student’s t-test |  | t_(22)_=0.241 | p=0.812 |
|  |  |  |  |  |  |  |  |
|  | Dp(10)2Yey | WT = 12 Mut = 12 | contact time with novel vs. familiar OiP associations [sec] | ANOVA | genotype | F_(1,22)_=0.705 | p=0.410 |
|  |  |  |  |  | object | F_(1,22)_=9.324 | p=**0.006** |
|  |  |  |  |  | genotype x object | F_(1,22)_=0.129 | p=0.722 |
|  |  |  | mean discrimination ratio vs. chance (0.5) | one sample t-test |  | WT: t_(11)_=2.769  Mut: t_(11)_=2.126 | WT: **p=0.018** Mut: p=0.057 |
|  |  |  | mean discrimination | Student’s t-test |  | t_(22)_=0.163 | p=0.872 |

**Table S4. Statistical comparison of 12-13-month-old Dp1Tyb, Dp(17)3Yey and Dp(10)2Yey cohorts and their WT littermates during the test phase of the OiP task.** Significant results (p<0.05) highlighted in bold. Mut = Mutant; WT = Wildtype.

| **Task** | **Strain** | **Sample size** | **Measure** | **Statistical test** | **Factor** | **test value** | **p value** |
| --- | --- | --- | --- | --- | --- | --- | --- |
|  |  |  |  |  |  |  |  |
| Loc at 12-13 months (test phase) | Dp1Tyb | WT: 11  Mut: 11 | contact time with objects in old vs. novel location [sec] | ANOVA | genotype | F_(1,20)_=0.036 | p=0.851 |
|  |  |  |  |  | object | F_(1,20)_=24.809 | **p<0.001** |
|  |  |  |  |  | genotype x object | F_(1,20)_=0.569 | p=0.460 |
|  |  |  | mean discrimination ratio vs. chance (0.5) | one sample t-test |  | WT: t_(10)_=5.114  Mut: t_(10)_=5.320 | WT: **p<0.001**  Mut: **p<0.001** |
|  |  |  | mean discrimination | Student’s t-test |  | t_(20)_=1.094 | p=0.287 |

**Table S5. Statistical comparison of 12-13-month-old Dp1Tyb and their WT littermates during the test phase of the Loc task.**
Significant results (p<0.05) highlighted in bold. Mut = Mutant; WT = Wildtype.

| **Task** | **Strain** | **Sample size** | **Measure** | **Statistical test** | **Factor** | **Test value** | **p value** |
| --- | --- | --- | --- | --- | --- | --- | --- |
|  |  |  |  |  |  |  |  |
| NOR at 18-20 months (sample phases) | Dp1Tyb | WT = 9 Mut = 6 | contact time with objects in 2 identical sample phases [sec] | ANOVA | genotype | F_(1,13)_=5.485 | **p=0.036** |
|  |  |  |  |  | sample phase | F_(1,13)_=2.225 | p=0.160 |
|  |  |  |  |  | genotype x sample phase | F_(1,13)_=5.678 | **p=0.033** |
|  |  |  |  |  | simple main effects of genotype | S1: F_(1,13)_=2.992  S2: F_(1,13)_=1.436 | S1: **p=0.012**  S2: p=0.326 |
|  |  |  |  |  | simple main effects of sample phase | WT: F_(1,13)_=3.063  Mut: F_(1,13)_=0.575 | WT: **p=0.018**  Mut: p>0.999 |
|  |  |  |  |  |  |  |  |
|  | Dp(10)2Yey | WT = 9 Mut = 11 | contact time with objects in 2 identical sample phases [sec] | ANOVA | genotype | F_(1,18)_=5.018 | **p=0.038** |
|  |  |  |  |  | sample phase | F_(1,18)_=3.691 | p=0.071 |
|  |  |  |  |  | genotype x sample phase | F_(1,18)_=0.003 | p=0.960 |
|  |  |  |  |  |  |  |  |
| NOR & OiP at 18-20 months (sample phases) | Dp(17)3Yey | WT = 9 Mut = 12 | contact time with objects in 2 identical sample phases [sec] | ANOVA | genotype | F_(1,19)_=0.521 | p=0.479 |
|  |  |  |  |  | sample phase | F_(1,19)_=1.820 | p=0.193 |
|  |  |  |  |  | genotype x sample phase | F_(1,19)_=0.041 | p=0.842 |
|  |  |  |  |  |  |  |  |
| Loc at 18-20 months (sample phases) | Dp(10)2Yey | WT = 9  Mut = 11 | Contact time with objects in 2 identical sample phases [sec] | ANOVA | genotype | F_(1,18)_=0.023 | p=0.881 |
|  |  |  |  |  | sample phase | F_(1,18)_=0.797 | p=0.384 |
|  |  |  |  |  | genotype x sample phase | F_(1,18)_=0.236 | p=0.633 |

**Table S6. Statistical comparison of 18-20-month-old Dp1Tyb, Dp(17)3Yey and Dp(10)2Yey cohorts and their WT littermates during sample phases of recognition memory tasks.** Significant results (p<0.05) highlighted in bold. Mut = Mutant; WT = Wildtype.

| **Task** | **Strain** | **Sample size** | **Measure** | **Statistical test** | **Factor** | **test value** | **p value** |
| --- | --- | --- | --- | --- | --- | --- | --- |
|  |  |  |  |  |  |  |  |
| NOR at 18-20 months (test phase) | Dp1Tyb | WT = 9 Mut = 6 | contact time with novel vs. familiar objects [sec] | ANOVA | genotype | F_(1,13)_=11.592 | **p=0.005** |
|  |  |  |  |  | object | F_(1,13)_=91.970 | **p<0.001** |
|  |  |  |  |  | genotype x object | F_(1,13)_=11.224 | **p=0.005** |
|  |  |  |  |  | simple main effects of genotype | nov: F_(1,13)_=16.658  fam: F_(1,13)_=4.065 | nov: **p=0.001**  fam: p=0.065 |
|  |  |  |  |  | simple main effects of object | WT: F_(1,13)_=104.658  Mut: F_(1,13)_=16.223 | WT: **p<0.001**  Mut: **p=0.001** |
|  |  |  | mean discrimination ratio vs. chance (0.5) | one sample t-test |  | WT: t_(8)_=10.170  Mut: t_(5)_=5.120 | WT: **p<0.001**  Mut: **p=0.004** |
|  |  |  | mean discrimination ratio | Student’s t-test |  | t_(13)_=0.713 | p=0.488 |
|  |  |  |  |  |  |  |  |
|  | Dp(17)3Yey | WT = 9 Mut = 12 | contact time with novel vs. familiar objects [sec] | ANOVA | genotype | F_(1,19)_=0.016 | p=0.899 |
|  |  |  |  |  | object | F_(1,19)_=9.718 | **p=0.006** |
|  |  |  |  |  | genotype x object | F_(1,19)_=0.386 | p=0.542 |
|  |  |  | mean discrimination ratio vs. chance (0.5) | one sample t-test |  | WT: t_(8)_=3.695  Mut: t_(11)_=2.930 | WT: **p=0.006**  Mut: **p=0.014** |
|  |  |  | mean discrimination ratio | Student’s t-test |  | t_(19)_=0.544 | p=0.593 |
|  |  |  |  |  |  |  |  |
|  | Dp(10)2Yey | WT = 9 Mut = 11 | contact time with novel vs. familiar objects [sec] | ANOVA | genotype | F_(1,18)_=0.660 | p=0.427 |
|  |  |  |  |  | object | F_(1,18)_=9.889 | **p=0.006** |
|  |  |  |  |  | genotype x object | F_(1,18)_=2.104 | p=0.164 |
|  |  |  | mean discrimination ratio vs. chance (0.5) | one sample t-test |  | WT: t_(8)_=4.500  Mut: t_(10)_=0.058 | WT: **p=0.002**  Mut: p=0.955 |
|  |  |  | mean discrimination | Student’s t-test |  | t_(18)_=2.206 | **p=0.041** |

**Table S7. Statistical comparison of 18-20-month-old Dp1Tyb, Dp(17)3Yey and Dp(10)2Yey cohorts and their WT littermates during the test phase of the NOR task.** Significant results (p<0.05) highlighted in bold. Mut = Mutant; WT = Wildtype.

| **Task** | **Strain** | **Sample size** | **Measure** | **Statistical test** | **Factor** | **test value** | **p value** |
| --- | --- | --- | --- | --- | --- | --- | --- |
|  |  |  |  |  |  |  |  |
| OiP at 18-20 months (test phase) | Dp(17)3Yey | WT = 8 Mut = 12 | contact times with novel vs. familiar OiP associations [sec] | ANOVA | genotype | F_(1,18)_=0.168 | p=0.687 |
|  |  |  |  |  | object | F_(1,18)_=10.030 | **p=0.005** |
|  |  |  |  |  | genotype x object | F_(1,18)_=0.011 | p=0.919 |
|  |  |  | mean discrimination ratio vs. chance (0.5) | one sample t-test |  | WT: t_(7)_=3.426  Mut: t_(11)_=2.513 | WT: **p=0.011**  Mut: **p=0.029** |
|  |  |  | mean discrimination ratio | Student’s t-test |  | t_(18)_=0.134 | p=0.895 |

**Table S8. Statistical comparison of 18-20-month-old Dp(17)3Yey and their WT littermates during the test phase of the OiP task.**
Significant results (p<0.05) highlighted in bold. Mut = Mutant; WT = Wildtype.

| **Task** | **Strain** | **Sample size** | **Measure** | **Statistical test** | **Factor** | **test value** | **p value** |
| --- | --- | --- | --- | --- | --- | --- | --- |
|  |  |  |  |  |  |  |  |
| Loc at 18-20 months (test phase) | Dp(10)2Yey | WT = 9 Mut = 11 | contact times with objects in familiar vs. novel locations [sec] | ANOVA | genotype | F_(1,18)_=0.376 | p=0.547 |
|  |  |  |  |  | object | F_(1,18)_=14.910 | **p=0.001** |
|  |  |  |  |  | genotype x object | F_(1,18)_=0.409 | p=0.530 |
|  |  |  | mean discrimination ratio vs. chance (0.5) | one sample t-test |  | WT: t_(8)_=2.323  Mut: t_(10)_=3.501 | WT: **p=0.049**  Mut: **p=0.006** |
|  |  |  | mean discrimination ratio | Student’s t-test |  | t_(18)_=0.581 | p=0.568 |

**Table S9. Statistical comparison of 18-20-month-old Dp(10)2Yey and their WT littermates during the test phase of the Loc task.**
Significant results (p<0.05) highlighted in bold. Mut = Mutant; WT = Wildtype.

| **Measure** | **Strain** | **Age** | **Sample size** | **Statistical test** | **Group mean** | **Test value** | **p value** |
| --- | --- | --- | --- | --- | --- | --- | --- |
|  |  |  |  |  |  |  |  |
| Body weight [g] | Dp1Tyb | 8-12 months | WT = 11 Mut = 12 | Student’s t-test | WT=35 (±2.88)  Mut=32g (±2.85) | t_(21)_= 2.339 | **p=0.029** |
|  |  | 20 months | WT = 9 Mut = 7 |  | WT=41g (±4.50)  Mut=33g (±1.65) | t_(14)_= 4.189 | **p<0.001** |
|  |  |  |  |  |  |  |  |
|  | Dp(17)3Yey | 8-12 months | WT = 12 Mut = 12 | Student’s t-test | WT=37g (±2.66)  Mut =38g (±2.23) | t_(22)_=1.165 | p=0.256 |
|  |  | 20 months | WT = 9 Mut = 12 |  | WT=36g (±4.64)  Mut=37g (±3.12) | t_(19)_=0.274 | p=0.787 |
|  |  |  |  |  |  |  |  |
|  | Dp(10)2Yey | 8-12 months | WT = 12 Mut = 12 | Student’s t-test | WT=37g (±5.07)  Mut=35g (±4.98) | t_(22)_=0.731 | p=0.473 |
|  |  | 20 months | WT = 9  Mut = 11 |  | WT=38g (±4.52)  Mut=39g (±3.89) | t_(18)_=0.401 | p=0.693 |

**Table S10. Statistical comparison of body weight in Dp1Tyb, Dp(17)3Yey and Dp(10)2Yey cohorts and their WT littermates.**Significant results (p<0.05) highlighted in bold. Mut = Mutant; WT = Wildtype.

| **Measure** | **Strain** | **Age** | **Sample size** | **Statistical test** | **Group mean** | **Test value** | **p value** |
| --- | --- | --- | --- | --- | --- | --- | --- |
|  |  |  |  |  |  |  |  |
| Size of hippocampal dissection [mg] | Dp1Tyb | 21 months | WT = 6 Mut = 5 | Student’s t-test | WT=15.64 (±3.57)  Mut=15.34 (±1.92) | t_(9)_=0.173 | p=0.866 |
|  |  |  |  |  |  |  |  |
|  | Dp(17)3Yey | 21 months | WT = 9 Mut = 12 | Student’s t-test | WT=19.32 (±2.37)  Mut =17.76 (±2.72) | t_(19)_=1.375 | p=0.185 |
|  |  |  |  |  |  |  |  |
|  | Dp(10)2Yey | 14 months | WT = 9 Mut = 11 | Student’s t-test | WT=20.22 (±1.51)  Mut=19.55 (±3.49) | t_(19)_=0.587 | p=0.564 |
|  |  | 22 months | WT = 6  Mut = 10 |  | WT=19.58 (±3.43)  Mut=21.14 (±3.06) | t_(14)_=0.944 | p=0.361 |

**Table S11. Statistical comparison of hippocampal dissection size in Dp1Tyb, Dp(17)3Yey and Dp(10)2Yey cohorts and their WT littermates.**No significant results (p>0.05). Mut = Mutant; WT = Wildtype.

| **Measure** | **Strain** | **Age** | **Sample size** | **Statistical test** | **Measure** | **Test value** | **p value** |
| --- | --- | --- | --- | --- | --- | --- | --- |
|  |  |  |  |  |  |  |  |
| HPC protein expression by immunoblots | Dp1Tyb | 21 months | WT = 6 Mut = 5 | Student’s t-test | GluN1 | t_(5)_=0.394 | p=0.709 |
|  |  |  |  |  | GluA1 | t_(9)_=2.371 | **p=0.042** |
|  |  |  |  |  | pGluA1(S845) | t_(9)_=3.075 | **p=0.013** |
|  |  |  |  |  | GluA1 cyt | t_(9)_=0.391 | p=0.705 |
|  |  |  |  |  | GluK5 | t_(9)_=1.049 | p=0.322 |
|  |  |  |  |  | PSD95 | t_(9)_=2.985 | **p=0.015** |
|  |  |  |  |  | DYRK1A cyt | t_(9)_=3.440 | **p=0.007** |
|  |  |  |  |  |  |  |  |
|  | Dp(17)3Yey | 21 months | WT = 9 Mut = 12 |  | GluN1 | t_(19)_=0.483 | p=0.634 |
|  |  |  |  |  | GluA1 | t_(19)_=0.188 | p=0.853 |
|  |  |  |  |  | GluK5 | t_(19)_=0.394 | p=0.698 |
|  |  |  |  |  | PSD95 | t_(19)_=0.886 | p=0.387 |

**Table S12. Statistical comparison of hippocampal protein abundance in Dp1Tyb and Dp(17)3Yey and their WT littermates.**

Significant results highlighted in bold (p>0.05). All proteins were measured in HPC synaptosomes, except for proteins noted with cyt that were measured in HPC cytosol. Mut = Mutant; WT = Wildtype.

| **Measure** | **Strain** | **Age** | **Sample size** | **Statistical test** | **Measure** | **Test value** | **p value** |
| --- | --- | --- | --- | --- | --- | --- | --- |
|  |  |  |  |  |  |  |  |
| Frontal cortex protein expression by immunoblots | Dp1Tyb | 21 months | WT = 6 Mut = 5 | Student’s t-test | GluA1 | t_(9)_=0.726 | p=0.486 |
|  |  |  |  |  | PSD95 | t_(9)_=0.571 | p=0.582 |
|  |  |  |  |  |  |  |  |
|  | Dp(10)2Yey | 22 months | WT = 5 Mut = 11 |  | Gluk5 | t_(14)_=0.092 | p=0.928 |
|  |  |  |  |  | PSD95 | t_(14)_=1.184 | p=0.256 |

**Table S13. Statistical comparison of frontal cortex protein abundance in Dp1Tyb and Dp(10)2Yey and their WT littermates.**

No significant results (p>0.05). All proteins were measured in frontal cortex synaptosomes. Mut = Mutant; WT = Wildtype.

| **Measure** | **Strain** | **Age** | **Sample size** | **Statistical test** | **Measure** | **Factor** | **Test value** | **p value** |
| --- | --- | --- | --- | --- | --- | --- | --- | --- |
|  |  |  |  |  |  |  |  |  |
| HPC protein expression by immunoblots | Dp(10)2Yey | 14 months | WT = 9 Mut = 11 | Kruskal-Wallis test | GluN2A |  | χ2_(3)_=0.940 | p=0.816 |
|  |  | 22 months | WT = 6  Mut = 10 |  |  |  |  |  |
|  |  |  |  |  |  |  |  |  |
|  |  |  |  | Kruskal-Wallis test | GluN2B |  | χ2_(3)_=2.095 | p=0.553 |
|  |  |  |  |  |  |  |  |  |
|  |  |  |  |  |  |  |  |  |
|  |  |  |  | Kruskal-Wallis test | pGluN2B(Y1472) |  | χ2_(3)_=2.434 | p=0.487 |
|  |  |  |  |  |  |  |  |  |
|  |  |  |  |  |  |  |  |  |
|  |  |  |  | ANOVA | GluN1 | genotype | F_(1,32)_=2.743 | p=0.107 |
|  |  |  |  |  |  | age | F_(1,32)_=2.495 | p=0.124 |
|  |  |  |  |  |  | genotype x age | F_(1,32)_=2.460 | p=0.127 |
|  |  |  |  | ANOVA | GluA1 | genotype | F_(1,32)_=0.453 | p=0.506 |
|  |  |  |  |  |  | age | F(_1,32)_=0.030 | p=0.864 |
|  |  |  |  |  |  | genotype x age | F(_1,32)_=2.150 | p=0.152 |
|  |  |  |  | ANOVA | GluK5 | genotype | F_(1,32)_=5.960 | **p=0.020** |
|  |  |  |  |  |  | age | F_(1,32)_=0.206 | p=0.653 |
|  |  |  |  |  |  | genotype x age | F_(1,32)_=5.556 | **p=0.025** |
|  |  |  |  |  |  | simple main effects of genotype | 14 mo: F_(1,32)_=0.004  22mo: F_(1,32)_=10.117 | 14 mo: p=0.949  22 mo: **p=0.003** |
|  |  |  |  |  |  | simple main effects of age | WT: F_(1,32)_=3.334  Mut: F_(1,32)_=2.222 | WT: p=0.077  Mut: p=0.146 |
|  |  |  |  |  | PSD95 | genotype | F_(1,32)_=0.929 | p=0.342 |
|  |  |  |  |  |  | age | F_(1,32)_=2.277 | p=0.141 |
|  |  |  |  |  |  | genotype x age | F_(1,32)_=4.638 | **p=0.039** |
|  |  |  |  |  |  | simple main effects of genotype | 14 mo: F_(1,32)_=0.821  22 mo: F_(1,32)_=4.270 | 14 mo: p=0.372  **22 mo: p=0.047** |
|  |  |  |  |  |  | simple main effects of age | WT: F_(1,32)_=0.175  Mut: F_(1,32)_=8.233 | WT: p=0.678  **Mut: p=0.007** |

**Table S14. Statistical comparison of hippocampal protein abundance in Dp(10)2Yey and their WT littermates at two different ages.**

Significant results highlighted in bold (p>0.05). All proteins were measured in HPC synaptosomes. Mut = Mutant; WT = Wildtype.
